# ﻿*Vacciniumbangliangense*, a new species of Ericaceae from limestone areas in Guangxi, China

**DOI:** 10.3897/phytokeys.194.81018

**Published:** 2022-04-11

**Authors:** Yu-Song Huang, Shi-Yue Nong, Xing-Kang Li, Gao Xie, Yi-Hua Tong

**Affiliations:** 1 Guangxi Key Laboratory of Plant Functional Phytochemicals and Sustainable Utilization, Guangxi Institute of Botany, Guangxi Zhuang Autonomous Region and the Chinese Academy of Sciences, Guilin, Guangxi, 541006, China Guangxi Institute of Botany, Guangxi Zhuang Autonomous Region and the Chinese Academy of Sciences Guilin China; 2 College of life Sciences, Guangxi Normal University, Guilin, Guangxi, 541004, China Guangxi Normal University Guilin China; 3 Guangxi Bangliang Gibbon National Nature Reserve Administration, Baise, Guangxi, 533800, China Guangxi Bangliang Gibbon National Nature Reserve Administration Baise China; 4 College of Tourism & Landscape Architecture, Guilin University of Technology, Guilin, Guangxi, 541006, China Guilin University of Technology Guilin China; 5 Key Laboratory of Plant Resources Conservation and Sustainable Utilization, South China Botanical Garden, Chinese Academy of Sciences, Guangzhou, Guangdong, 510650, China South China Botanical Garden, Chinese Academy of Sciences Guangzhou China; 6 Center of Conservation Biology, Core Botanical Gardens, Chinese Academy of Sciences, Guangzhou, Guangdong, 510650, China Core Botanical Gardens, Chinese Academy of Sciences Guangzhou China

**Keywords:** limestone flora, morphology, new species, south-western Guangxi, *Vaccinium*

## Abstract

*Vacciniumbangliangense*, a new species from limestone areas in Guangxi, China, is described and illustrated. It is morphologically most similar to *V.pseudotonkinense* and *V.sciaphilum* in having small and dense obovate leaf blades with a retuse apex, hairy young branches and calyx and campanulate corollas, but can be distinguished from them by the distance of basal gland from petiole, the length of peduncle, pedicle and filaments, the indumentum of calyx tube and corolla and the existence of apical glands on calyx lobes. A table to distinguish the new species from other morphologically similar *Vaccinium* species, as well as colour plates of comparison of key characters, is also provided.

## ﻿Introduction

The genus *Vaccinium* L. contains 450–500 species distributed worldwide (Fang 1991; Fang and Stevens 2005; Vander Kloet and Dickinson 2009). Currently, there are 98 species of *Vaccinium* known from China (Fang 1986; Fang and Stevens 2005; Tong and Xia 2015; Tong et al. 2018, 2020, 2021a, b, 2022). As one of the most biodiverse regions of China, Guangxi has 27 species and two varieties of the genus *Vaccinium*, including four endemic species, viz. *V.damingshanense* Y.H. Tong & N.H. Xia, *V.napoense* Y.H. Tong & N.H. Xia, *V.crassivenium* Sleumer and *V.cuspidifolium* C.Y. Wu & R.C. Fang (Qin and Liu 2010; Tang 2011; Huang et al. 2015; Tong and Xia 2015; Tong et al. 2018, 2020).

During fieldwork in Bangliang Gibbon National Nature Reserve of Guangxi in June 2021, we discovered a special flowering plant of *Vaccinium* never recorded from Guangxi with the characteristics of inflorescence being shortly racemose, axillary or borne on leafless old stems, peduncle being very short or 4–5 mm long and corolla being broadly campanulate, yellowish-green or tinged reddish. After consulting Flora of China (Fang and Stevens 2005) and other relevant literature (Dop 1930; Pham 1999; Nguyen 2005; Newman et al. 2007; Qin and Liu 2010; Tang 2011; Tong and Xia 2015; Watthana 2015; Tong et al. 2020), as well as comparisons amongst this unknown species and its morphologically most similar species, based on herbarium specimens including types, we confirmed that this species is new to science, which is described and illustrated below.

## ﻿Materials and methods

Field surveys have been conducted in flowering and fruiting phases at the type locality. Measurements and assessments of morphological characters were based on the living plants in the wild and the specimens gathered from the type locality. Type specimens were deposited in the herbaria of South China Botanical Garden (IBSC) and Guangxi Institute of Botany (IBK). The comparisons amongst this unknown species, *V.sciaphilum* C.Y. Wu and *V.pseudotonkinense* Sleumer were based on the descriptions from protologues and the examination of herbarium specimens or photos of specimens (including types) at IBK, IBSC, KUN and P (Sleumer 1941; Fang and Wu 1987). The habitat information and threatened factors were recorded during field surveys. The assessment of threatened status of the new species is based on the IUCN Red List of Threatened Species Categories and Criteria and Guidelines for using the IUCN Red List Categories and Criteria (IUCN 2012; IUCN Standards and Petitions Committee 2022).

## ﻿Taxonomic treatment

### ﻿ Vaccinium bangliangense

Taxon classificationPlantaeEricalesEricaceae

Y.S. Huang & Y.H. Tong sp. nov.

75D5DB9D-0C26-579D-9F27-E754C438A255

urn:lsid:ipni.org:names:77296991-1

**Figure 1.**
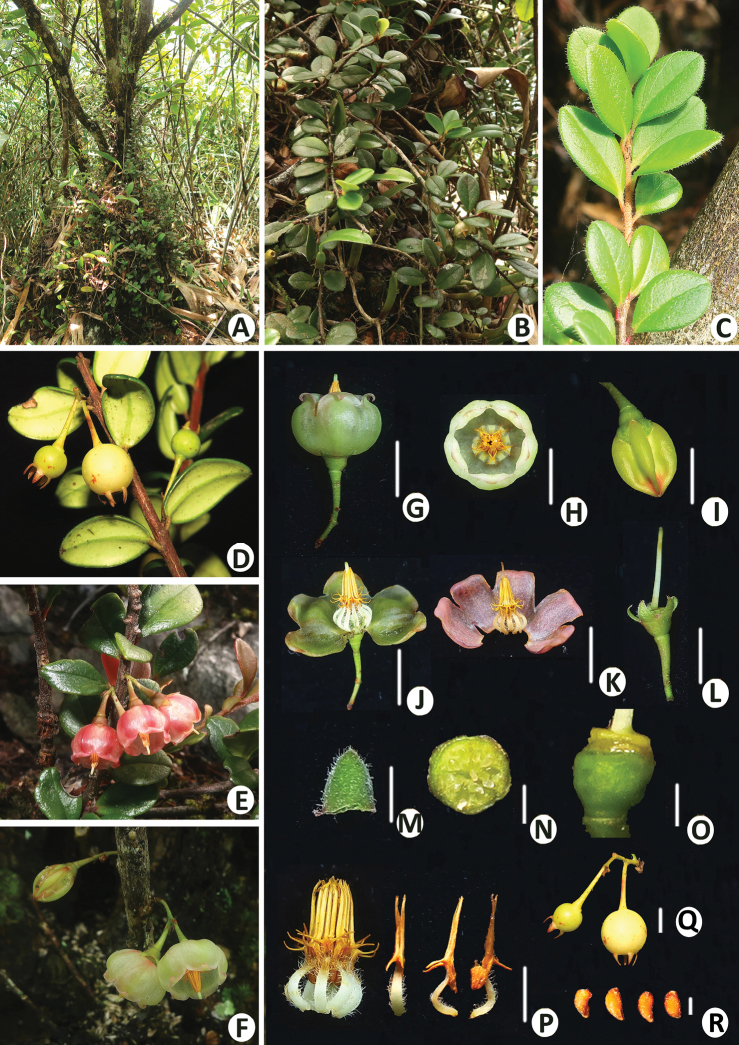
*Vacciniumbangliangense***A** habitat **B** habit **C** young branch, showing the leaves with a sparsely villous margin **D** fruiting branch **E** flowering branch with tinged reddish flowers **F** inflorescence borne on leafless old stem with yellowish-green flowers **G** flower (lateral view) **H** flower (vertical view) **I** flower bud **J, K** flowers with opened corolla **L** flower with corolla and stamens removed **M** bracteole **N** transection of ovary **O** calyx tube and disc **P** androecium and adaxial (left), lateral (middle) and abaxial (right) view of a stamen **Q** infructescence **R** seeds. Scale bars: 5 mm (**G–I, P, Q**); 1 mm (**M–O, R**).

**Figure 2.**
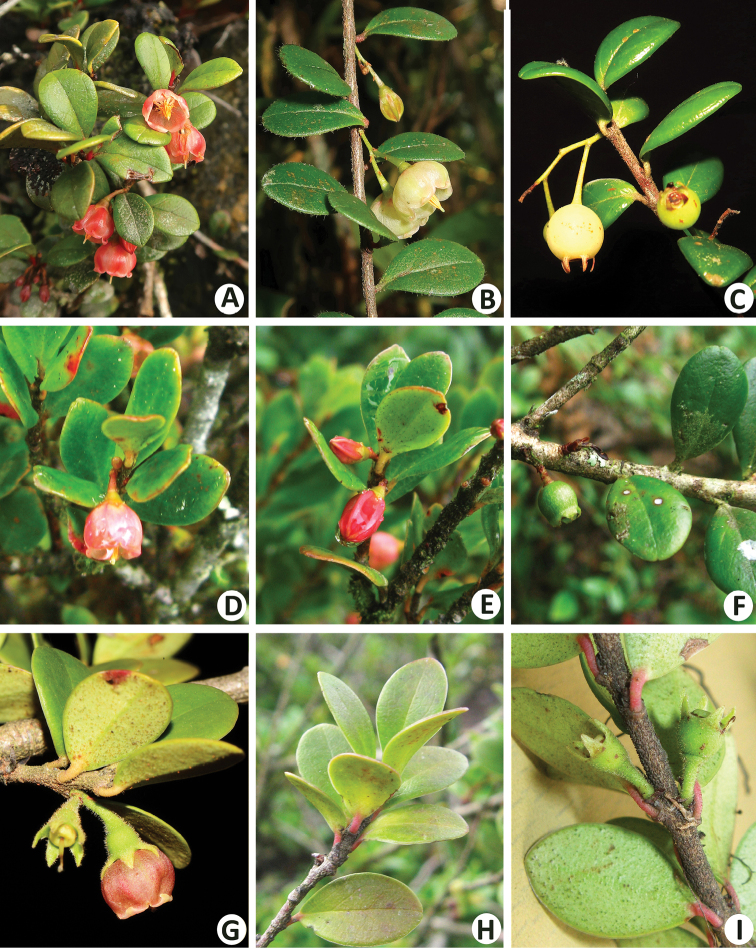
**A–C***Vacciniumbangliangense***A** flowering branch with tinged reddish flowers **B** flowering branch with yellowish-green flowers **C** fruiting branch **D–F***V.pseudotonkinense***D** flowering branch with opened flower **E** flowering branch with unopened flower **F** fruiting branch **G–I***V.sciaphilum***G** flowering branch **H** leafy branch **I** fruiting branch. All by Yi-Hua Tong, except **G** by Xin-Xin Zhu.

**Figure 3.**
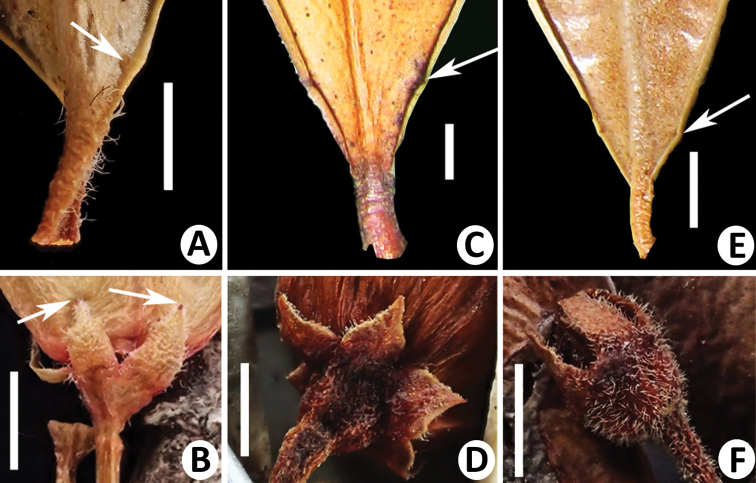
**A, B***Vacciniumbangliangense***A** basal gland **B** calyx, arrows showing apical glands **C, D***V.pseudotonkinense***C** basal gland **D** calyx, showing lobes with a ciliate and glandular margin **E, F***V.sciaphilum***E** basal gland **F** calyx. Scale bars: 2 mm.

**Figure 4.**
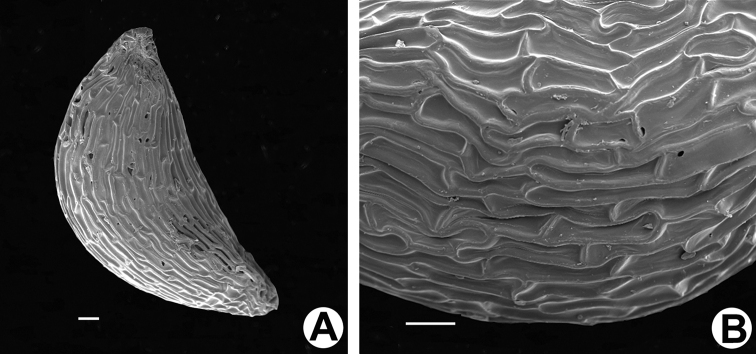
*Vacciniumbangliangense***A** SEM micrograph of seed **B** elongated cells of testa. Scale bars: 100 μm.

Figs 1 , 2A–C , 3A, B , 4

#### Diagnosis.

*Vacciniumbangliangense* Y.S. Huang & Y.H. Tong belongs to V.sectionConchophyllumSleumer (1941) and is morphologically similar to *V.pseudotonkinense* Sleumer and *V.sciaphilum* C.Y. Wu in having small and dense obovate leaf blades with a retuse apex, hairy young branches and calyx and campanulate corollas, but can be distinguished from the former by basal glands on leaf blade margin at 0.3–0.8 mm (vs. 2.6–4 mm, Fig. 3C) distance from petiole, inflorescence with very short peduncle or up to 5 mm long (vs. very short, Fig. 2E), calyx lobes with ciliolate margin and a gland at apex (vs. with ciliate and glandular margin and without a gland at apex, Fig. 3D) and, from the latter, by inflorescence with very short peduncle or up to 5 mm long (vs. very short, Fig. 2G), longer (5–7 mm vs. ca. 3 mm) and glabrous (vs. densely pubescent, Fig. 2G) pedicel, glabrous or sparsely villous (vs. densely hispid) calyx tube, glabrous or sparsely pubescent (vs. densely hispid, Fig. 3F) calyx lobes with a gland at apex (vs. without a gland at apex, Fig. 2I) and densely villous (vs. glabrous) filaments. A detailed morphological comparison amongst the three species is summarised in Table 1.

**Table 1.** A morphological comparison of key characters of *Vacciniumbangliangense*, *V.sciaphilum* and *V.pseudotonkinense*.

Character	*V.bangliangense*	*V.pseudotonkinense*	*V.sciaphilum*
Distance of basal gland from petiole	0.3–0.8 mm	2.6–4 mm	0.8–1.7 mm
Inflorescence	2-flowered or solitary, peduncle very short or 3–5 mm long	solitary, sometimes 2-flowered, peduncle very short	solitary, sometimes 2-flowered, peduncle very short
Pedicel	5–7 mm long, glabrous	ca. 4 mm long, glabrous	ca. 3 mm long, densely pubescent
Calyx tube	glabrous or sparsely villous	glabrous or sparsely pubescent	densely hispid
Calyx lobes	glabrous or sparsely pubescent abaxially, margin ciliolate, apex with a gland	glabrous or sparsely pubescent abaxially, margin ciliate and glandular, apex without a gland	densely hispid abaxially, margin ciliate, apex without a gland
Corolla lobes	pubescent abaxially at apex	glabrous	glabrous
Filaments	densely villous, ca. 2 mm long	sparsely pilose, ca. 1 mm long	glabrous, ca. 1 mm long
Ratio of anther thecae and tubule	1:2	1:2	1:1

#### Type.

China. Guangxi Zhuang Autonomous Region: Baise City, Jingxi City, Renzhuang Town, Bangliang protection station, campsite of Huitun, 850 m a.s.l., 2 June 2021, *S.Y. Nong & P. Yang ZYA00199* (holotype: IBSC!; isotypes: IBK!, IBSC!).

#### Description.

Small evergreen shrubs, 10–20 cm tall, usually epilithic, sometimes epiphytic on tree trunks. Stem ascending, with long creeping rhizomes. Roots with bead-like swellings 1–3 cm in diam. Young branches brownish-red, grey when older, densely white villous, glabrescent. Leaves dense; petiole 1–2 mm long, red, adaxially sparsely pubescent, glabrescent, abaxially glabrous; blades obovate or broadly obovate, 1–1.7 × 0.6–1.2 cm, leathery, adaxially near margin sparsely villous when young, glabrescent, abaxially sparsely brown glandular hispidulous, base cuneate, basal gland 1 per side, at 0.3–0.8 mm distance from petiole, margin entire, revolute, apex obtuse, slightly retuse; mid-vein impressed adaxially, raised abaxially; lateral veins 2–4 pairs, flat and inconspicuous or impressed adaxially, raised abaxially. Inflorescence shortly racemose, 2-flowered or solitary, axillary or borne on leafless old stems; peduncle very short or 3–5 mm long, base with several bracts, glabrous or sparsely white villous on distal part; bracts ovate, 0.5–1 mm long, ca. 0.5 mm wide, margin ciliolate, apex glandular; pedicel 5–7 mm long, glabrous, thickening towards the apex, articulate with the calyx tube; bracteoles 2, adnate to 1–3 mm above the pedicle base, triangular-ovate, ca. 1 × 0.5 mm, margin ciliolate, apex glandular; calyx tube green or purple green, obconical, ca. 1.5 × 2 mm, glabrous or sparsely villous; calyx limb divided nearly to the base; lobes 5, triangular-ovate, ca. 2 × 1.5 mm, both surfaces glabrous or sparsely pubescent abaxially, margin ciliolate, apex glandular; corolla yellowish-green or tinged reddish, broadly campanulate, ca. 9 × 5 mm, both surfaces glabrous, 5-lobed; lobes triangular-ovate, apical part reflexed, apex acute, pubescent abaxially, glabrous adaxially; stamens 10, 5.5–6 mm long; filaments white, tinged reddish in reddish flowers, flat, incurved, ca. 2 mm long, densely villous; anthers yellow, 4–5 mm long, thecae ca. 1.5 mm long, tubules 3–3.5 mm long, with 2 spurs at the base abaxially, spurs ca. 1.3 mm long, interlocked (that is: the spurs on antesepalous stamens extending laterally outside of antepetalous anthers and strongly overlapping with spurs of next antesepalous stamens, those on antepetalous anthers strongly hooked outward below spurs of antesepalous stamens); disc yellowish, annular, glabrous; style greenish, tinged reddish in reddish flowers, cylindrical, ca. 6.5 mm long, glabrous, stigma truncate; ovary pseudo-10-locular, each locule with several ovules. Berry globose, ca. 8 mm in diam., glabrous, greenish when young, white when mature, fruiting calyx persistent, narrowly triangular-ovate and slightly inflexed; seeds reniform, ventrally compressed, 1.5–2 mm long, testa brownish, cells elongated, with thickened anticlinal walls.

#### Phenology.

*Vacciniumbangliangense* was observed flowering from May to June and fruiting from August to October (and up to January of the following year in indoor cultivated plants).

#### Etymology.

The specific epithet is derived from the type locality, Bangliang Gibbon National Nature Reserve of Guangxi, China. The Chinese name is given as “ 邦亮越橘 (pinyin: bāng liàng yuè jú)”.

#### Distribution and habitat.

Thus far, *Vacciniumbangliangense* was found only in Bangliang Gibbon National Nature Reserve of Guangxi, China. It usually grows on rocks of limestone hillside or peak at an elevation from 850–900 m, sometimes on the trunks of *Pistaciaweinmanniifolia* J. Poisson ex Franchet (Anacardiaceae). The slope direction is to the south and the slope gradient is ca. 30°. The tree layer is up to 8 m tall with a canopy cover of 70% and the shrub and herb layer covers are 85% and 20%, respectively. The associated species include *Quercusphillyreoides* A. Gray (Fagaceae), Sinosideroxylonpedunculatum(Hemsl.)H. Chuangvar.pubifolium H. Chuang (Sapotaceae), *Ardisiapseudocrispa* Pit. (Primulaceae), *Schefflerapesavis* R. Vig. (Araliaceae), *Tetradiumcalcicola* (Chun ex C.C. Huang) T.G. Hartley (Rutaceae), *Sageretiacamelliifolia* Y.L. Chen & P. K. Chou (Rhamnaceae), *Paraboeaswinhoei* (Hance) B.L. Burtt (Gesneriaceae), *Boniaamplexicaulis* (L.C. Chia et al.) N.H. Xia (Poaceae), *Bulbophyllumandersonii* (Hook. f.) J.J. Smith (Orchidaceae) etc.

#### Conservation status.

*Vacciniumbangliangense* has only been found in Bangliang Gibbon National Nature Reserve of Guangxi, China. As a new species, more subpopulations of *V.bangliangense* could probably be found in similar habitats of surrounding limestone areas in the future. However, wild surveys have been conducted for more than ten years in the area where the new species was found. Only two subpopulations were found in the protected region with a total of fifteen individuals and seven of these are mature. Based on the current data, its population size is very small, and the area of occupancy (AOO) is restricted. According to Guidelines for Using the IUCN Red List Categories and Criteria (IUCN Standards and Petitions Committee 2022), the conservation status of *V.bangliangense* should be assessed as Critically Endangered (CR), based on criteria D of (IUCN 2012).

#### Additional specimens examined

**(paratypes).** China. Guangxi Zhuang Autonomous Region: Baise City, Jingxi City, Renzhuang Town, Bang Liang protection station, 900 m a.s.l., 2 June 2021, *S.Y. Nong & P. Yang NSY2021060201* (IBK); Guilin City, cultivated in Botany Garden of Guilin, collected from the same locality as above, 16 January 2022, *Y.S. Huang 2022011601* (IBK).

## ﻿Discussion

In Guangxi, another species of V.sect.Conchophyllum, namely *V.triflorum* Rehder, is also somewhat similar to this new species in the small and dense leaves, short racemes and campanulate corollas, but can be readily distinguished by its thickly leathery and elliptic or obovate-elliptic leaf blades with a strongly rugose adaxial surface. The two species also have allopatric distribution in Guangxi: *V.triflorum* is distributed in Huanjiang County, north Guangxi, while *V.bangliangense* occurs in Jingxi County, southwest Guangxi.

Two kinds of flower colour of *Vacciniumbangliangense* were observed in the wild: the plants growing in shaded habitat always bear inflorescences with yellowish-green flowers and longer peduncles (Figs 1F, 2B), while inflorescences with tinged reddish flowers and shorter peduncles (Figs 1E, 2A) are normally found in sunlit habitat. In addition, the leaf blades of plants growing in shady habitat are thinner and with sparsely white villous margin (Fig. 2B), while those of plants growing in sunlit habitat are thicker and with less hairy or glabrous margins (Fig. 2A). It is speculated that these variations on flower colour, peduncle length and texture and indumentum of leaf blades may be caused by different light intensity.

*Vacciniumbangliangense* is a small shrub with a beautiful tree form and thus an excellent species for landscaping. It is adaptive to limestone areas and has important application value in limestone mountain greening. The first author once inserted one branch of this species into clear water for more than 3 months and surprisingly found that it grew new roots, which indicates that this plant is relatively easy to be cultivated.

## Supplementary Material

XML Treatment for Vaccinium bangliangense
